# The Constrained Disorder Principle Accounts for the Variability That Characterizes Breathing: A Method for Treating Chronic Respiratory Diseases and Improving Mechanical Ventilation

**DOI:** 10.3390/arm91050028

**Published:** 2023-09-09

**Authors:** Ofek Adar, Adi Hollander, Yaron Ilan

**Affiliations:** 1 Faculty of Medicine, Hebrew University, Jerusalem P.O. Box 1200, Israel; ofek.adar@mail.huji.ac.il (O.A.); adi.hollandr@gmail.com (A.H.); 2 Department of Medicine, Hadassah Medical Center, Jerusalem P.O. Box 1200, Israel

**Keywords:** constrained disorder, variability, energy, mitochondria, artificial intelligence, digital health, T_TOT_: V_T_ respiratory time, EEV_L_: end-expiratory lung volume

## Abstract

**Highlights:**

**What are the main findings?**

- The constrained disorder principle (CDP) defines systems by their inherent disorder bounded by variable boundaries.

**What is the implication of the main finding?**

- The present paper describes the mechanisms of breathing and cellular respiration, focusing on their inherent variability and how the CDP accounts for the variability in breathing and respiration.
- The article describes using CDP-based artificial intelligence platforms to augment the respiratory process’s efficiency and treat respiratory diseases.

**Abstract:**

Variability characterizes breathing, cellular respiration, and the underlying quantum effects. Variability serves as a mechanism for coping with changing environments; however, this hypothesis does not explain why many of the variable phenomena of respiration manifest randomness. According to the constrained disorder principle (CDP), living organisms are defined by their inherent disorder bounded by variable boundaries. The present paper describes the mechanisms of breathing and cellular respiration, focusing on their inherent variability. It defines how the CDP accounts for the variability and randomness in breathing and respiration. It also provides a scheme for the potential role of respiration variability in the energy balance in biological systems. The paper describes the option of using CDP-based artificial intelligence platforms to augment the respiratory process’s efficiency, correct malfunctions, and treat disorders associated with the respiratory system.

## 1. Introduction

Variability characterizes the respiration process at the cellular level and in the lungs. It manifests randomly and lacks a regular pattern [1,2]. Quantum effects linked to randomness may underlie some of the mechanisms involved in respiration [3]. Living organisms are defined by the constrained disorder principle (CDP), and complex systems function by their inherent disorder within variable parameters [4].

This paper reviews the variations at different levels of the respiration process and how CDP accounts for them. The paper describes the potential of CDP-based artificial intelligence (AI) systems for correcting diseased states that impact respiration. We selected the relevant studies describing the role of variability in cellular respiration and lung functions and the relevant studies on CDP-based AI’s use to improve organ malfunctions.

### 1.1. The Constrained Disorder Principle Defines Biological Processes

Variability characterizes the proper function of biological systems. It underlies the function of multiple processes in the genome, microtubules, cellular functions, and whole organs [5,6,7,8,9,10,11,12,13,14,15,16,17,18,19]. The CDP defines living organisms by their degree of disorder bounded by dynamic, continuously changing random boundaries [4]. The principle elucidates the multiple processes that underlie systems’ adaptability, flexibility, and energy use [20]. The CDP can correct systems’ malfunctions because diseased states evolve from low or high degrees of variability. It serves as a platform for second-generation AI systems designed to augment systems’ efficiency [21,22,23].

### 1.2. The Constrained Disorder Principle Accounts for the Stochasticity in Respiration

Per the CDP, variability, when kept within borders, defines living organisms and is essential for proper respiration [4]. Variability in breathing exists in the resting state and characterizes all organisms, supporting the CDP-based concept that variability is mandatory for proper respiration. It raises the issue of why variability occurs under what seems to be stable and unchanged conditions in the same individual and the benefits of having a degree of variability for the system.

Variability is necessary for assisting with continuous internal and external perturbations in the respiratory systems and is part of the adaptability and flexibility of complex systems [4]. What looks like stable conditions is a sum of numerous continuously changing perturbations mandating a degree of variability for maintaining a proper function. However, one would expect it to take specific patterns of response. The variability seems random and lacks fixed patterns for whole lung function, and cellular respiration implies that it may be part of a CDP-based energy efficiency mechanism of complex systems [20,24]. This mechanism requires dynamic boundaries to the variability, which adapts to the changes in the internal and external environments [4].

In normal subjects, breathing patterns vary in rate, rhythm, depth, and duration [25,26,27]. Mechanical and chemical changes within the respiratory system alter variability [28,29,30]. Breathing control involves complex mechanisms that balance the opposing goals of efficiency, redundancy, responsiveness, and stability [31]. This variability is maintained by a central neural mechanism and feedback loops between lung vagal sensory receptors and arterial chemoreceptors [23]. Respiratory rate, tidal volume, and airflow profiles vary due to inputs, positive and negative feedback loops, internal pacemakers, and non-linear interactions between components [31].

Variability analysis is a method to describe the differences between data groups [32]. Two groups of time-series data can have the same mean values but different variability. Breathing pattern variability can be quantified using quantitative methods, including coefficients of variation and Poincaré plots [31,32,33]. Using the Poincaré plot analysis, breathing patterns are dynamically analyzed breath-by-breath in real time [34].

Breathing-related physiological variables, such as tidal volume (VT) and respiratory rate, exhibit significant breath-to-breath variability [2,35,36]. Cycle-by-cycle variations of respiratory physiological variables are highly reproducible in individual subjects when measured using long-range correlations [2]. The output of a control system reflects the underlying control system’s fluctuations as they act on feedback loops [37].

The model output generated a cyclic pattern with breath-to-breath variations that mimicked VT’s properties when noise was added to the neural network model of the brain respiratory oscillator [38]. Cycle-by-cycle variability impacts the functioning of cells in the respiratory system [2]. Sleep, pulmonary diseases, hypoxia, and anxiety disorders affect breathing variability [2]. Variability also occurs in resting states, suggesting that it is inherent to normal pulmonary function.

The variability and correlation properties of spontaneous breathing in humans were investigated in seated healthy subjects. α represents the scaling exponent of a power-law fluctuation function. For a random process, α has a value of 0.5. A positively correlated signal shows large fluctuations following large fluctuations. A signal with small fluctuations following large fluctuations has a value between 0 and 0.5 and is considered anti-correlated [2]. Breath-by-breath, the VT, respiratory time (TTOT), and other breathing pattern parameters were computed. Regarding minute ventilation and respiratory rate, VT, TTOT, and other breathing variables showed values between 0.60 and 0.71 [2].

The brain’s respiratory center is one of the regulatory systems for fluctuations in breathing parameters; it receives input from other brain centers. Except for the end-expiratory lung volume (EEVL), which is related to tissue viscoelasticity, all respiratory variables are correlated with the brain [2]. Correlated fluctuations originate in the brain, but periphery input is necessary to maintain the oscillations [39]. The oscillator’s output would be a deterministic cyclic pattern with constant frequency and amplitude without fluctuations in the inputs to the neural oscillator [40]. While the correlated properties of EEVL are influenced by the brain oscillator and viscoelastic memory of the tissues, breathing patterns are influenced by the brain and neural noise [2,41].

Oscillators are non-linear networks of neuron groups, so lung and other brain area fluctuations can produce cycle-by-cycle variations in the output, which may be influenced by respiratory muscles [41]. Stretch-sensitive adherent cells in the respiratory system are affected by cycle-by-cycle fluctuations in tidal volume and respiratory time [42]. As a result of this variability, respiratory system cells adapt [2].

A reduction in the active brain control of respiratory variables occurs during sleep [43]. Brainstem catecholamine (CA) neurons manifest an arousal-state-dependent activity pattern and participate in the control of breathing while modulating the processing of sensory information [44,45]. CA neurons are involved in central respiratory chemoreception, which boosts breathing frequency and lowers breathing variations during rapid eye movement (REM) sleep [46].

Per the CDP, respiratory variability is inherent to normal cellular respiration and lung function and characterizes brain centers, lungs, and respiratory cells. Although it can adapt to the changing environment, variability also characterizes the resting state, implying that the respiratory system requires it for proper function [4].

The key to understanding how the inherent variability in breathing and respiration contributes to the system’s function and efficiency is to be determined. Another unresolved issue is to what extent this variability is personalized and to what extent learning and systems’ history impact the variability.

### 1.3. The Regulation of Cellular Respiration and Electrons Transport

As a result of cellular respiration, free energy can be released from carbohydrate, fat, and protein energy substrates in a controlled manner [47,48]. While the sequence seems to follow a particular ordered path, variability underlies many of these processes.

In the cytosol, glycolysis occurs, while in the mitochondria, the citric acid cycle occurs, along with oxidative phosphorylation [49]. It requires glucose, adenosine triphosphate (ATP), and NAD+; its products are ATP and H_2_O [50]. Oxidative phosphorylation involves the electron transport chain and chemiosmosis [51]. Electrons travel along the transport chain’s components through redox reactions. The energy of these reactions release is captured as a proton gradient, which makes ATP and is termed chemiosmosis [24,52].

In addition to proton leakage, electron leakage from electron transport chain complexes uncouples membrane voltage from ATP production. If electrons leak prematurely, they pass directly to oxygen rather than through oxygen to form water, causing superoxide to form, which can be toxic to cells [53]. This process suggests a more complicated regulatory mechanism involving a degree of variability.

There are several regulatory steps along the respiration pathway. An enzyme that catalyzes the pathway’s first committed step is the main target for regulating biochemical processes [53]. When an enzyme is active, a specific step proceeds; if it is inactive, the step may not occur or will be slowed. The binding of regulatory molecules ATP, ADP, and NADH controls respiration enzymes at the allosteric sites. Increased ATP level implies that a cell has sufficient ATP, leading to inhibition of the respiratory system [54].

A vital control point is a phosphofructokinase (PFK). Adenosine monophosphate (AMP) is a positive regulator of PFK, while ATP is a negative regulator [55]. By converting pyruvate to acetyl CoA, pyruvate dehydrogenase controls the acetyl CoA entering citric acid. When ATP and NADH are present, this enzyme is less active; when ADP is present, it is more active. Pyruvate dehydrogenase is also activated by its substrate, pyruvate, and inhibited by its product, acetyl CoA [56].

Pyruvate dehydrogenase regulates citric acid cycle entry. Additional regulatory points include the release of carbon dioxide molecules and the production of the first two NADH molecules of the cycle. ADP and ATP inhibit isocitrate dehydrogenase, while NADH activates it. ATP, NADH, and several other molecules, including succinyl CoA, inhibit α-ketoglutarate dehydrogenase [57,58].

Additional regulatory mechanisms are the oxygen delivery to tissues, regulation of oxygen binding to the heme moiety of cytochrome oxidase by NO, availability of nutrient metabolism to generate NADH and FADH_2,_ and the overall cellular energy state defined by the ratio of ATP/ADP [57,58,59].

By oxidative phosphorylation, mitochondria coordinate energy demands with energy production in the cytosol. Calcium signals between the cytosol and matrix regulate this process. Transfer of Ca^2+^ into the mitochondria signals an increased energy demand. Upregulating citric acid cycle enzymes, increasing respiration, and increasing ATP synthesis increases energy provision by oxidative phosphorylation [60,61]. Changes in mitochondrial morphology impact the bioenergetic state, and changes in bioenergetics result in altered morphology [62].

These control mechanisms underlie the ordered process of cellular respiration but do not explain the inherent variability of the processes.

### 1.4. The Constrained Disorder Principle Accounts for Variability in Cellular Respiration

Variation in respiratory functions serves as a mechanism for systems’ flexibility and adaptation to environmental changes [63]. The respiratory system acclimates over time to the environment by adjusting its enzyme capacities. Adaptation is the genetic divergence of the properties of the respiratory system [63]. Environmental factors induce variation in mitochondrial efficiency that impacts performance.

Living organisms adapt to local environmental conditions through natural selection of genetic variation. Genetic variation in mitochondrial efficiency and oxygen consumption is relevant for energy metabolism. Over time, mitochondria produce ATP at different rates in different subjects, populations, and environments within the same individual [64,65,66,67]. Throughout an individual’s lifetime, the degree of energy coupling derived from oxidation varies across tissues, among individuals, and over time [68,69].

Measuring ATP production from oxygen consumption is inaccurate because the amount produced per unit of oxygen consumed varies significantly [70]. Because of the inherent variability in the link between oxidation and phosphorylation and the amount of ATP generated per molecule of oxygen consumed by mitochondria (P/O ratio), oxygen consumption is not a reliable marker of energy metabolism. Diet and temperature affect the P/O ratio within and among subjects. Due to tissue-specific regulation of mitochondria, mitochondrial function and P/O ratio differ significantly among tissues within the same subject [71,72]. The variability of the P/O ratio impacts an organism’s performance. Reducing the P/O ratio is energetically costly but provides advantages via reduced production of reactive oxygen species [73,74].

The relationship between the P/O ratio and performance is not causative [75]. An optimal P/O ratio and oxygen consumption rate are shaped by extrinsic (food availability and temperature) and intrinsic variables (genotype, hormones, and substrate mobilization) [73]. A population, individual, and adaptive strategy for maximizing mitochondrial energy efficiency and operating at the lowest oxidative cost is necessary [73]. It is possible to expect an increase in the P/O ratio during periods of high energy demand, even if this results in an increase in reactive oxygen species (ROS) [76].

Variation in the P/O ratio involves proton leakage across the membrane, contributing to the drop in the electrochemical potential (Δp) and oxygen consumption, independent of ATP production [77,78]. Several factors affect mitochondrial membrane conductance, including phospholipid fatty acids and mitochondrial carrier proteins, such as the uncoupling protein (UCP) and the adenine nucleotide transporter [79]. The Δp is affected by the active transport across the inner membrane of anions (ADP^3−^ and ATP^4−^), cations (Ca^2+^), and metabolites (aspartate and glutamate) [73,80].

Temperature affects ATP production due to an increase in the H^+^ conductance of the inner membrane. At higher temperatures, more H+ is shunted away from ATP synthase, reducing P/O [81]. Low temperatures reduce mitochondrial coupling in brown adipose tissue, contributing to thermogenesis [82]. Acclimation to cold temperatures increases oxygen consumption but does not change the coupling of ATP production to oxygen consumption [83].

Stress compensates for lowered respiratory ATP production efficiency with higher respiratory rates [84]. Respiratory supply and demand changes accompany stress and mask the effects of lowered efficiency [85]. Oxidative phosphorylation improves respiration in low-oxygen environments. A study of fish populations with different temperature regimes and their gene expression of mitochondrial-encoded oxidative phosphorylation subunits identified seven transcripts showing increased expression levels that differ with changing temperatures, improving respiratory efficiency [86].

By reducing food intake, the P/O ratio increases, reducing ATP synthesis costs and energy substrate requirements [66,87]. As a result of diet, phospholipid properties of the inner mitochondrial membrane are altered, affecting the P/O ratio. Unsaturated fats increase protons’ permeability and mitochondrial proton leak, altering the P/O ratio [88].

Evolution was expected to maximize the P/O ratio for improved resource usage. However, natural selection did not maximize mitochondrial efficiency due to the generation of ROS. The variation in the P/O ratio may be because of the ATP production trade-off against ROS generation that results from a flow of electrons through the mitochondrial ETC [73,89,90,91,92]. Reducing the P/O ratio may slow aging via a reduction in ROS production [93]. By lowering mitochondrial respiration, partial pressure of oxygen build-up increases ROS production and ATP synthesis and accelerates respiration and electron flow through the ETC [94].

A cell’s ability to generate ATP determines its growth [95]. Growth efficiency varies between subjects depending on mitochondrial function. In individuals with higher growth efficiencies, UCP expression is reduced, proton leak is reduced, and P/O ratios are higher [96,97].

In plants, respiratory variability results from genetic, developmental, and environmental factors [63]. It optimizes respiration’s energetic and biosynthetic performance under changing conditions [63]. Physiological supply and demand conditions determine the rate at which substrates enter and leave the respiratory system. The flexibility of the process is reflected in variations in how carbon substrates are metabolized. Although substrate levels do not directly affect respiratory enzymes, they can indirectly stimulate respiration. A tissue’s respiration rate is regulated by respiratory enzymes whose regulatory properties are targeted by signaling based on energy status [98,99,100]. By engaging alternate routes and increasing the permeability of the mitochondrial inner membrane to protons, every ATP-producing reaction in respiration can be bypassed, resulting in a variation in ATP yield [101].

The inherent variability of the respiratory system is a challenge for modeling the regulatory control of the system.

### 1.5. Tunneling in Redox Reactions Implies That Variability Underlies Respiration at the Atomic Level and Is a Manifestation of Quantum Effects

While the sequences of cellular respiration and some of its variability are well determined, the classical approach cannot explain the atomic events and their regulation. From a thermodynamic perspective, a cell is a semi-open system that allows energy to enter and waste to leave [102,103]. If a cell’s energy levels fall, it disrupts the balance of order and disorder. It is because it has too much or lacks sufficient disorder, affecting the cell’s function and leading to cellular death [103]. According to the CDP, the cell requires energy to maintain order and disorder [24]. It implies that energy is required to maintain dynamic boundaries for systems’ function.

There is randomness at the core of the quantum world of atoms and particles [104]. Calculating probabilistic quantum wave functions entails randomness playing a role in this effect [105]. Tunneling, coherence, and entanglement are linked to DNA, which is the blueprint for respiratory chain proteins; it follows that quantum physics is linked with diseases of the respiratory chain [3].

During a redox reaction, electrons are transferred between two chemical entities simultaneously: oxidation (loss of electrons) and reduction (gain of electrons) [106]. Molecular pumps, concentration gradients across membranes, and energy-rich metabolites are all regulated by redox reactions. Alterations in redox balance contribute to aging and disease progression as the genome adapts to environmental challenges [107,108,109].

Proton transfer is crucial in several enzyme-catalyzed reactions, which involve the movement of protons between molecules and may involve quantum-mechanical tunneling, whereby a particle passes through an energy barrier rather than gathering energy to ‘climb’ over it [110]. Proton (hydrogen) tunneling plays a role in numerous enzyme reactions [110,111]. The proton-coupled electron transfer process involves long-range electron tunneling, and hydrogen tunneling is widespread in biological processes, including DNA repair, photosynthesis, cellular homeostasis, and cell death, being required for complex chemical transformations [112,113].

By connecting ROS partitioning and cellular bioenergetics, quantum effects bridge the atomic and cellular levels [114]. Coherent electron spin dynamics influence ROS production. Reduced flavoenzymes formed spin-correlated radical pairs during molecular oxygen activation in cell culture. Cellular superoxide and hydrogen peroxide ROS products are altered by oscillating magnetic fields, indicating coherent singlet-triplet mixing. The orientation dependence of magnetic stimulation involves changes in ROS levels, increasing mitochondrial respiration and glycolysis rates [114]. The radical pair mechanism determines how quantum effects affect ROS production. Electron-nuclear hyperfine interactions, internal magnetic interactions, and applied magnetic fields control spin dynamics [115].

An electron transfer activates oxygen in reduced flavoenzymes, resulting in magnetically sensitive ROS formation [116]. Spin-correlated radical pairs are formed between flavin semiquinone (FADH^•^) and superoxide (O_2_^•−^) [117]. Coherent evolution between the singlet and triplet states of FADH^•^:O_2_^•−^ radical pairs determines the products of the reaction [118]. ROS production is differentially affected at parallel excitation, indicating increased mitochondrial respiration [119,120,121,122].

The data support the notion that quantum mechanics is essential and that randomness plays a role in the proper respiration process [123]. The CDP applies to quantum effects and thus defines both classical and quantum effects in respiration [4].

### 1.6. Altered Variability in Lung Diseases

The CDP implies that chronic lung diseases are associated with deviations in breathing pattern variability [124,125,126]. Altered variability in physiological and cellular characteristics in different pulmonary diseases can predict and possibly prevent clinical deterioration [127]. Additionally, variability can be a valuable tool for diagnosis and treatment adjustments in those patients.

Respiratory variability is lower in chronic obstructive pulmonary disease (COPD) than in healthy patients. It may be due to lung and chest wall mechanics changes or neural adjustments in breathing control [31]. COPD reduces the variation in inter-breathing intervals that characterize normal breathing and is influenced by metabolic demands [128]. Patients with COPD have more regular breath-to-breath fluctuations than those without COPD, and the increased regularity is correlated with the severity of the disease. Compared to healthy controls, patients with COPD need to breathe faster to compensate, which increases oxygen demand [128].

Patients with restrictive lung disease display breathing variability significantly smaller than healthy patients [129]. The variability is significant for tidal volume and expiratory time. It increases dyspnea in response to slight variations from the average resting tidal volume. During lung disease of infancy, non-REM sleep, and highly demanding cognitive tasks, breathing variability is reduced [31]. Variability in tidal breathing parameters is a risk factor for developing subsequent respiratory morbidity in preterm infants [129]. During the first year of life, preterm infants with low variability in tidal breathing parameters were more likely to be re-hospitalized [129].

In contrast to patients with COPD and interstitial lung disease (ILD), asthmatics’ respiration variability increases with the disease’s severity [31]. The quantitative variability of tidal breathing parameters in children with asthma was increased and improved but not normalized following bronchodilators [130]. Hypoxia, hypertension, and anxiety disorders increase the variability of older adults during complex arithmetic tasks [31]. It is also affected by the use of different anesthetics and sedation drugs.

Non-invasive pulmonary function tests (PFTs) identify daily and day-to-day variability. Peak expiratory flow (PEF) variability measures are reliable predictors of loss of asthma control and response to treatment [131,132,133]. The quantitative variability of minute ventilation and tidal volume is higher in COPD patients than in age- and sex-matched controls, and sighs are significantly reduced in COPD patients [134]. Another method to evaluate variability in airway resistance is using the forced oscillation technique (FOT) [135]. This method predicts asthma exacerbations [135,136]. In COPD, there is similar high day-to-day variability in FOT [137]. FOT variability is related to symptoms and precedes them by a few days and can detect changes before COPD exacerbation, enabling preemptive measures [138]. Patients with ILD show significant variability in forced vital capacity (FVC) during the day. This variability increases in patients with progressive disease compared to stable ones. FVC variability may be used in patients with ILD as a disease progression predictor [139].

Subjects receiving an endotoxin challenge, as well as postoperative and acutely ill patients, have reduced breathing pattern variability [140,141]. All organs, including the respiratory center, arterial chemoreceptors, lung vagal sensory receptors, and lung mechanics regulating breathing variability, are affected by endotoxin [142,143].

These data follow the CDP, implying that reduced or out-of-bounds high degrees of variability are associated with reduced systems’ efficiency.

### 1.7. Using the Constrained Disorder Principle-Based Platform for Augmenting Cellular Respiration and Improving Therapies for Chronic Respiratory Diseases

Variability characterizes the proper function of the respiratory system. Per the CDP, loss of variability or increased variability outside borders is associated with a diseased system [4]. Developing a CDP-based second-generation AI system, which implements algorithms based on variability, can correct system malfunctions and augment efficiency, increasing the variability in cases where it is too low or controlling it in cases where it is out of control. Based on the CDP, these measures are expected to improve clinical conditions by overcoming drug tolerance in patients with chronic lung conditions [4,144].

The algorithm can be implemented for the respiratory system to improve medication response in patients with chronic lung disorders who develop tolerance to therapies. By measuring the reaction to beta-agonists following bronchoconstriction with methacholine, it has been shown that regular beta-agonists use leads to tolerate their bronchodilator effects [145]. The bronchodilator response to salbutamol is reduced in patients taking formoterol due to tolerance. Despite increased bronchoconstriction and reduced FEV1, regular salbutamol attenuated the acute response to agonists. Increasing bronchoconstriction conferred increased susceptibility to the effects of bronchodilator tolerance [146,147].

The second-generation AI system is based on three levels. At the first level, the system implements variability in the output therapeutic regimen independent of the input. It provides a randomized medication regimen for an inhaler regarding dosage and administration time within a pre-defined approved range [21,22,23]. At the second level, a closed-loop system is implemented, wherein the system collects data on the output. The clinically meaningful endpoints are used as continuous inputs for the algorithm, altering the randomization to reach a better outcome. The algorithm personalizes the output based on the inputs it receives [4,19,21,22,23,144,148,149,150,151,152,153,154,155,156,157,158,159,160,161,162,163,164,165,166,167,168,169,170,171,172]. At the third level, the algorithm receives inputs from the user’s quantifications of variability signatures. These include measurements of the variability of different breathing parameters or variability in cytokines measurements. The algorithm uses large datasets from big data resources on patients with similar lung conditions while updating them and personalizing them to the individual patient [19,148,149,150,151,152,153,154,155,156,157,158,159,160,161,162,163,164,165].

**Figure 1:**
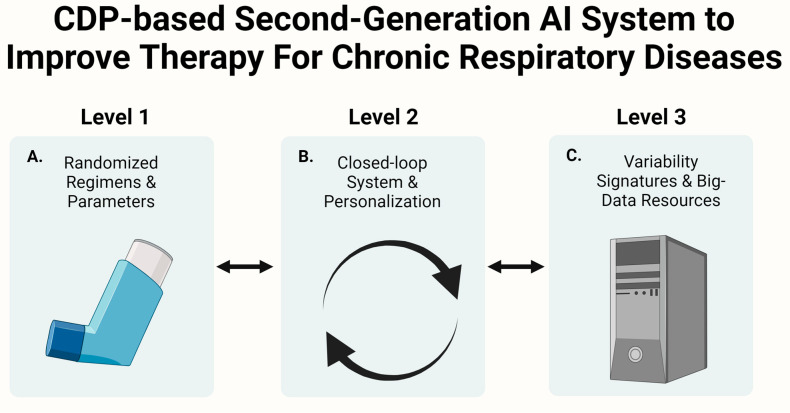
Overcoming tolerance to bronchodilators by implementing variability-based therapeutic regimens: The figure summarizes the design and function of a CDP-based second-generation AI system for medical treatment, specifically in the context of lung conditions. The system is based on three levels of operation: (**A**) The AI provides randomized medication regimens within a pre-defined range, aimed to overcome treatment tolerance. (**B**) The AI collects data on the output and uses clinically meaningful endpoints as inputs to personalize the output, which alters randomization to reach a better outcome. (**C**) The AI receives inputs from the patient’s quantified variability signatures and uses large datasets from big-data resources to further personalize the treatment for the individual patient. CDP, constrained disorder principle. AI, artificial intelligence. Created with Biorender.com.

Figure 1 shows a schematic presentation of the three steps for overcoming tolerance to bronchodilators by implementing variability-based therapeutic regimens.

### 1.8. The CDP-Based Platform for Improving Mechanical Ventilation and Assessment of Extubation Readiness

The CDP-based second-generation AI system can improve mechanical ventilation’s effectiveness, ease weaning from devices, and overcome drug tolerance in patients with chronic lung diseases by implementing variabilities.

Fixed breathing frequencies and monotonous tidal volumes characterize current ventilation methods. Using this non-physiological method of breathing ignores breathing variability and may damage the lungs, causing them to collapse and lowering the partial pressure of oxygen in the arteries to abnormally low levels [173]. To address this issue, ‘noisy ventilation’ was developed using biologically variable computer-controlled ventilators that incorporate noise to mimic natural variability. Opening collapsed alveoli without damaging them may be possible by occasional high pressures [174]. ‘Noisy ventilation’ implements breathing variability and improves oxygenation in animals with low mean airway pressure, comparable to minute ventilation [175].

Variable ventilation was tested in several preclinical studies. In a porcine model of oleic-acid-induced acute lung injury, animals received variable ventilation, which included changing breathing frequency in the range of 15–27 breaths/min and variable V_T_ or conventional ventilation at a rate of 20 breaths/min. There was a significant improvement in arterial oxygenation and lower shunt fraction in the variable ventilation group [176]. Compared to conventional mechanical ventilation, variable ventilation led to a twofold increase in bronchoalveolar lavage phospholipid surfactant levels and reduced alveolar protein content in guinea pigs [177]. If variable ventilation is combined with PEEP, recruitment is more sustained than monotonous controlled mechanical ventilation [178,179]. VT fluctuation at a frequency of 0.05 Hz improved oxygenation during variable ventilation in an experimental model of severe acute respiratory distress syndrome (ARDS) [42]. Variable ventilation may be further enhanced by deterministic resonance. Mechanical ventilation improves gas exchange and lung mechanics with varying VT in ARDS [180,181]. As a result, ventilator-induced lung injury (VILI) is reduced [182]. Variable ventilation facilitates uniform recruitment of the lungs without enlarging hyperaerated lung areas during acute lung injury [178,183].

During elective abdominal aortic aneurysmectomy, 41 patients were randomized to conventional or variable ventilation. With variable ventilation, arterial oxygenation and pulmonary compliance were higher with lower P_aCO2_ and dead-space ventilation [184]. The variable ventilation model also succeeded in patients with other lung injuries besides ARDS [185].

Both unnecessarily long extubation times and premature extubation are linked with adverse outcomes [186]. Assessment of extubation readiness for invasively ventilated patients is associated with a substantial failure rate [187,188]. Variability in breathing also serves as a predictor of successful extubation [189]. Altered respiratory rate variability and reduced complexity are reliable predictive tools for failed extubation in adults [190]. When applied to basic respiratory parameters, breathing variability predicts extubation failure, and comprehensive breathing variability indices improve the prediction. Based on continuous ventilation waveforms, breath-to-breath basic and comprehensive respiratory parameters were computed an hour before extubation [188]. During spontaneous breathing, intermittent deep-inflation or sigh breaths are characterized by large VT breaths (2–3 times standard VT) [191]. The sigh breath airs collapsed alveoli, improves full residual capacity (FRC), reduces pulmonary shunt, and resets the breathing pattern in excessive or unvarying breathing patterns [191]. Applying sigh breaths to ventilated hypoxemic patients improves ventilator-free days [192,193].

Weaning failure during mechanical ventilation is predicted by low respiratory variability. Patients who failed weaning trials had higher variability in spontaneous tidal volume and peak inspiratory flow [194]. In patients who failed to wean after cardiac surgery, the pattern of tidal volume, but not the respiratory rate, was more irregular due to weaning, and hemodynamic and autonomic nervous system changes occur [32]. In postoperative patients recovering from systemic inflammatory response syndrome (SIRS), breathing pattern variability was a weaning predictor [142]. For 30 min before weaning, tidal volume, total breath duration, inspiratory time, expiratory time, and peak inspiratory flow were monitored in mechanically ventilated SIRS patients. The five parameters’ average values of total breath duration, inspiratory time, expiratory time, and coefficient of variation were lower in the failure group, suggesting that SIRS failure groups were less likely to maintain breathing pattern variability due to reduced regulatory mechanisms [142]. After 60 min of spontaneous breathing before extubation, ventilated patients successfully separated from the ventilator showed greater breathing variability [189]. A CV of tidal volume/inspiratory time ≥ 19% and a CV of inspiratory time/respiratory period ≥ 10% were used to determine whether extubation was successful or unsuccessful [189]. A reduction in heart rate variability during spontaneous breathing trials (SBT) was associated with extubation failure, demonstrating the importance of variability in different organs to maintain proper function [190,195]. In contrast, high variability in breathing patterns in children was associated with failed extubation [196].

**Figure 2:**
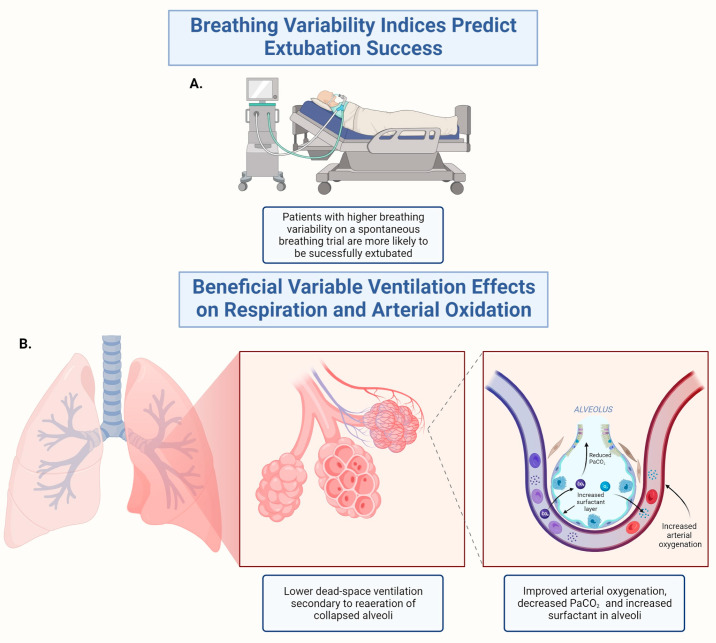
Inserting variability into mechanical ventilation to improve the efficacy of ventilation and ease the weaning process: Breathing variability serves as a predictor for successful extubation and improves the efficacy of mechanical ventilation. (**A**) Variability in breathing patterns, as measured by indices such as the CV of tidal volume/inspiratory time and CV of Inspiratory time/respiratory period, is an important predictor of extubation success or failure. (**B**) Variable ventilation was shown to be beneficial in animal models and in human patients, increasing arterial oxygenation, lowering shunt fraction and increasing pulmonary compliance. CV, coefficient of variability. Created with Biorender.com.

Figure 2 shows a schematic presentation of inserting variability into mechanical ventilation to improve the efficacy of ventilation and ease the weaning process.

## 2. Summary

Variability characterizes breathing and cellular respiration. Using variability by implementing disorder-based algorithms can improve the response to drugs in patients with chronic lung disorders and improve ventilation techniques. Personalization of variability-based algorithms and the linkage between the degree of variability and outcome are some of the future challenges faced by these platforms. Ongoing clinical studies are expected to shed light on the application of these measures in different clinical settings.

## Data Availability

All data are available on public domains.

## Abbreviations

Constrained disorder principle: CDP; artificial intelligence: AI; tidal volume: V_T_; respiratory time: T_TOT_; end-expiratory lung volume: EEV_L_; catecholamine: CA; rapid eye movement: REM; adenosine triphosphate: ATP; phosphofructokinase: PFK; adenosine monophosphate: AMP; reactive oxygen species: ROS; electrochemical potential: Δp; uncoupling protein: UCP; chronic obstructive pulmonary disease: COPD; interstitial lung disease: ILD; pulmonary function tests: PFTs; peak expiratory flow: PEF; forced oscillation technique: FOT; forced vital capacity: FVC; acute respiratory distress syndrome: ARDS; ventilator-induced lung injury: VILI; full residual capacity: FRC; systemic inflammatory response syndrome: SIRS; closing volume: CV; spontaneous breathing trials: SBT.
